# U-shaped association between myeloperoxidase levels and anxiety risk: a cross-sectional study in a Chinese population

**DOI:** 10.3389/fpubh.2025.1596844

**Published:** 2025-05-07

**Authors:** Junteng Zhou, Qihang Kong, Xiaojing Liu, Yan Huang

**Affiliations:** ^1^Health Management Center, General Practice Medical Center, West China Hospital, Sichuan University, Chengdu, China; ^2^Laboratory of Cardiovascular Diseases, Regenerative Medicine Research Center, West China Hospital, Sichuan University, Chengdu, China; ^3^Department of Cardiology, West China Hospital, Sichuan University, Chengdu, China; ^4^State Key Laboratory of Respiratory Health and Multimorbidity, West China Hospital, Sichuan University, Chengdu, China; ^5^Research Laboratory for Prediction and Evaluation of Chronic Diseases in the Elderly, National Clinical Research Center for Geriatric Diseases, West China Hospital, Sichuan University, Chengdu, China; ^6^General Practice Research Institute, West China Hospital, Sichuan University, Chengdu, China

**Keywords:** myeloperoxidase, anxiety, U-shaped association, diabetes mellitus, cross-sectional study, neuroinflammation

## Abstract

**Objective:**

This study investigates the association between myeloperoxidase (MPO) levels and anxiety risk in Chinese adults and explores potential effect modifiers, with implications for neuroinflammatory biomarker-guided anxiety prevention strategies.

**Methods:**

Using cross-sectional data from 30,418 adults undergoing routine health examinations (July 2020–June 2021), anxiety severity was assessed via the Self-Rating Anxiety Scale (SAS; score ≥ 50 as clinically relevant). Plasma MPO was quantified by ELISA. Multivariate logistic regression, restricted cubic splines (RCS), threshold effect analysis, and subgroup interactions were conducted to evaluate nonlinear associations.

**Results:**

A U-shaped relationship between MPO and anxiety risk was identified. In fully adjusted models, participants in the lowest (Q1: ≤29.77 ng/mL, OR = 1.15, 95% CI: 1.03–1.28, *p* = 0.01) and highest quintiles (Q5: ≥47.3 ng/mL, OR = 1.17, 95% CI: 1.05–1.31, *p* = 0.004) exhibited significantly elevated anxiety risks compared to the reference quintile (Q2: 29.8–34.7 ng/mL). RCS analysis confirmed a nonlinear association (*p* for nonlinearity < 0.01), with an inflection point at 30 ng/mL: below this threshold, each 1 ng/mL MPO increase reduced anxiety risk (OR = 0.982, CI: 0.970–0.994), while levels above it heightened risk (OR = 1.004, CI: 1.001–1.008). Diabetes mellitus significantly modified this relationship (*p*-interaction = 0.028), with diabetic individuals showing amplified risks at higher plasma MPO (Q5 OR = 1.84 vs. non-diabetic Q5 OR = 1.15).

**Conclusion:**

Plasma MPO demonstrates a U-shaped association with anxiety risk independent of cardiometabolic confounders. Diabetic individuals exhibit heightened susceptibility to MPO-related anxiety, suggesting synergistic neuroinflammatory pathways. Monitoring MPO may aid in risk stratification and personalized interventions, particularly in populations with diabetes.

## Introduction

Anxiety is one of the most prevalent mental health disorders worldwide, affecting millions of individuals across the globe (1). It can manifest at various life stages, including childhood, adolescence, and adulthood (2, 3). Anxiety is primarily characterized by excessive worry and associated behavioral disturbances, leading to both short-term and long-term distress and impairment (4). Studies have shown that anxiety is linked to various physiological symptoms, such as palpitations, dyspnea, and dizziness (4). Notably, anxiety is frequently observed in the progression of multiple diseases. For instance, substantial clinical data indicate an increased prevalence of anxiety among patients with multiple sclerosis (5). Similarly, a significant proportion of individuals with inflammatory bowel disease and gastroesophageal reflux disease also experience anxiety symptoms (6, 7). More importantly, anxiety is increasingly recognized as a critical contributor to disease pathogenesis (8). Evidence suggests that anxiety serves as a risk factor for cardiovascular diseases (9), Alzheimer’s disease (10), increased mortality in patients with implantable cardioverter-defibrillators (11), and acute exacerbations of chronic obstructive pulmonary disease (12). Given these associations, identifying reliable biomarkers of anxiety is crucial for the early diagnosis and personalized treatment of anxiety-related disorders.

As a neuropsychiatric disorder, anxiety is believed to result from a complex interplay of genetic, biological, psychological, and social factors (13). Among these, neuroinflammation is a critical phenotypic feature of anxiety disorders, and suppressing neuroinflammatory pathways has been shown to alleviate symptoms and promote recovery (14). Although various neuromodulation techniques have been employed to treat refractory psychiatric disorders, including anxiety, their efficacy varies among individuals, and reliable biological markers are lacking (15). Studies have demonstrated that inflammatory biomarkers are upregulated in anxiety-related disorders and are associated with individual differences in treatment response and adverse clinical outcomes (16). In patients with anxiety, pro-inflammatory cytokines such as IL-1α, IL-6, and IFN-*γ* are significantly elevated in serum and positively correlate with symptom severity (17). A study conducted in pediatric populations further suggested that inflammation-related oxidative dysfunction may contribute to the severity of anxiety symptoms (18). Additionally, heightened neuroinflammation may induce structural and functional alterations in anxiety-related brain regions, rendering individuals more susceptible to anxiety disorders (19). These findings underscore the potential of inflammatory biomarkers as valuable indicators for assessing anxiety-related pathophysiology and guiding treatment strategies.

Myeloperoxidase (MPO) is a heme-containing peroxidase expressed by myeloid cells, serving as a molecular mediator in the regulation of inflammatory responses (20). Studies have demonstrated that MPO can activate neuroinflammatory processes (21), and targeting MPO reduces neuroinflammation associated with X-linked dystonia-parkinsonism (22). Notably, MPO has been identified as a potential biomarker for Alzheimer’s disease, with implications for evaluating the therapeutic potential of its inhibitors (23). Additionally, MPO is considered a potential biomarker for depression following acute myocardial infarction, linking depressive symptoms to innate immune suppression (24). In bipolar disorder type I, impaired MPO activity has been associated with oxidative stress and inflammation (25), underscoring its role in psychiatric disorders. Although alterations in MPO activity have been observed in anxiety-like behaviors (26), a systematic investigation into the relationship between MPO and anxiety disorders remains lacking. Therefore, this study utilizes large-scale population data to explore the association between plasma MPO levels and anxiety risk, aiming to elucidate the biological significance and clinical relevance of MPO in anxiety. The findings of this study will provide new evidence for the role of neuroinflammation in anxiety disorders and contribute to biomarker-based anxiety risk prediction and personalized intervention strategies.

## Methods

### Study population

**Figure 1:**
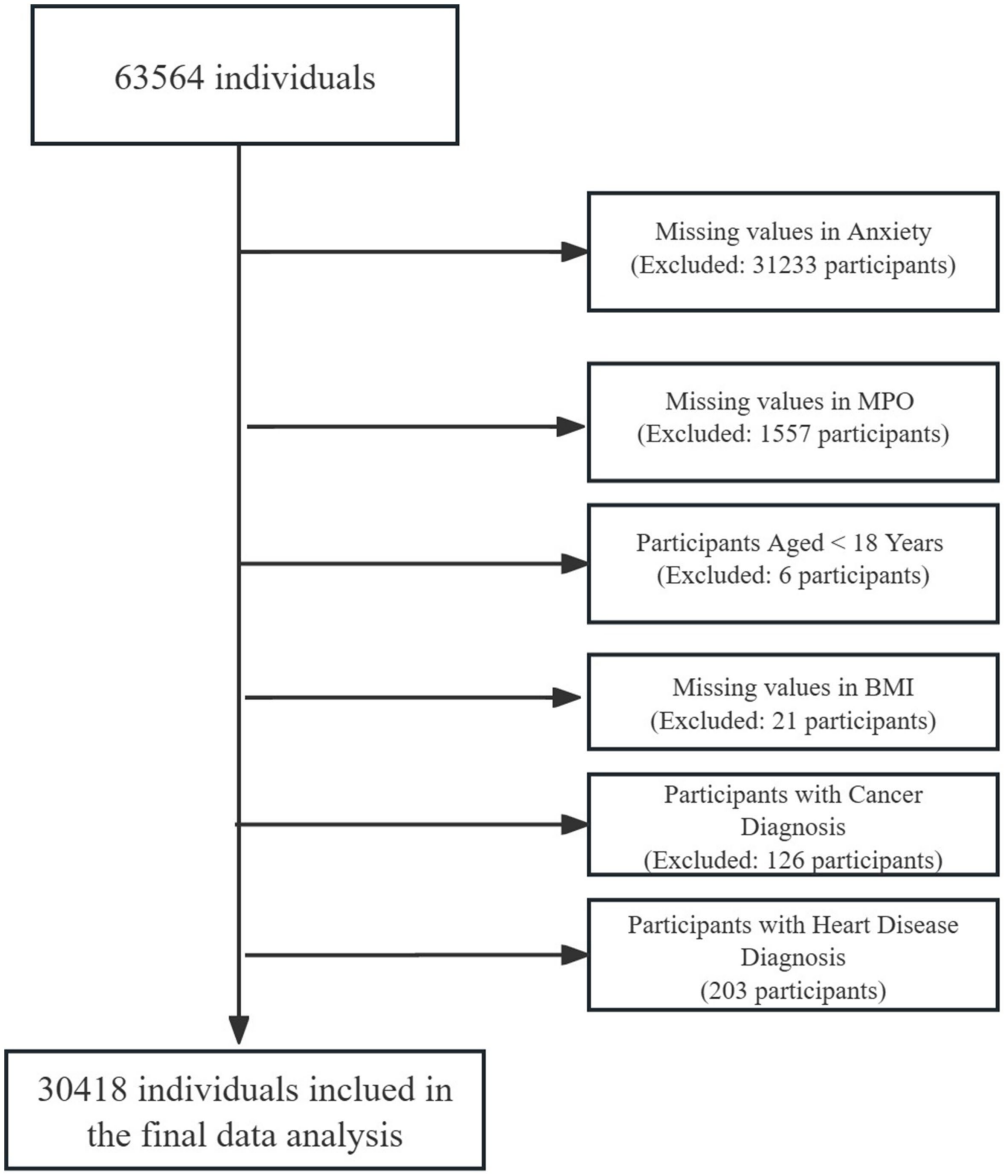
Flowchart of participant inclusion and exclusion criteria.

This cross-sectional analysis utilized data from 63,564 adults undergoing routine health examinations at the Health Management Center of West China Hospital, Sichuan University (July 2020–June 2021). Participants were required to meet the inclusion criteria of voluntary participation, age ≥ 18 years, and provision of informed consent. A sequential exclusion cascade refined the cohort: 31,233 individuals (49.0%) were excluded for missing anxiety status, followed by 1,557 (5.0% of remaining) with incomplete MPO measurements, 6 participants (0.02%) aged < 18 years, 21 (0.07%) lacking BMI data, 126 (0.41%) with cancer diagnoses, and 203 (0.66%) with heart disease. The final analytical sample included 30,418 participants with complete data for core variables: age, sex, BMI, smoking, alcohol use, hypertension, diabetes, hyperlipidemia, anxiety, and MPO (Figure 1). The characteristics of those individuals excluded due to exclusion criteria in the final analysis did not differ substantially from those included (Supplementary Table S1). Ethical approval was granted by the Ethics Committee of West China Hospital (No. 2018-303), with written informed consent obtained from all participants. The study utilized all available data from the health examination cohort meeting inclusion criteria (*n* = 63,564). After exclusions for missing data and clinical confounders, the final analytical sample (*n* = 30,418) ensured sufficient power for multivariable analyses. Post-hoc power analysis using the final sample (*n* = 30,418) indicated 99% power to detect an odds ratio ≥ 1.1 for anxiety risk across MPO quintiles (*α* = 0.05, baseline prevalence = 13%), calculated via the pwr package in R (v4.2.3). This approach aligns with observational studies prioritizing comprehensive data quality over prospective sample size calculations (27).

### Assessment of anxiety

Anxiety severity was evaluated using the 20-item Self-Rating Anxiety Scale (SAS), a widely recognized tool designed to quantify psychological and physiological symptoms associated with anxiety (28). Participants rated the frequency of specific emotional states (e.g., restlessness, tension) and physical manifestations (e.g., rapid heartbeat, dizziness) experienced over the previous 7 days. Each item employs a four-level response scale ranging from “rarely or never” (1 point) to “frequently or persistently” (4 points), with cumulative scores reflecting overall anxiety intensity. Consistent with culturally adapted diagnostic standards in China, individuals scoring 50 points or higher were classified as exhibiting clinically relevant anxiety symptoms. This threshold has been extensively applied in population-based studies to distinguish transient stress from pathological anxiety requiring intervention, ensuring alignment with regional epidemiological research practices. The scale’s design captures multidimensional aspects of anxiety, including cognitive, emotional, and somatic domains, thereby supporting holistic mental health assessments.

### Measurement of MPO and covariates

Plasma MPO concentrations were quantified using a commercial ELISA kit (EACHY, Suzhou, China) following standardized protocols as previously reported (29). Intra- and inter-assay coefficients of variation were maintained below 8% and 12%, respectively, with all samples analyzed in duplicate to ensure precision. Demographic covariates (age, sex, education, occupation) and lifestyle factors (smoking, alcohol use) were collected via structured questionnaires administered by trained interviewers. Smoking status was categorized as never (<100 lifetime cigarettes), former (quit ≥ 30 days), or current (active use). Alcohol consumption was classified as never (monthly or less), former (abstinence ≥ 6 months), or current (≥1 drink weekly). Clinical parameters including hypertension (systolic/diastolic blood pressure ≥ 140/90 mmHg or self-reported diagnosis), diabetes (fasting glucose ≥ 126 mg/dL, or HbA1c ≥ 6.5%, or self-reported diagnosis), and hyperlipidemia (triglycerides ≥ 150 mg/dL, LDL-C ≥ 130 mg/dL, or lipid-lowering medication use) were assessed through both biochemical assays and self-report. Anthropometric measurements (height, weight) were obtained using calibrated instruments, with BMI calculated as weight (kg)/height (m^2^).

### Statistical analysis

Continuous variables were presented as mean ± standard deviation and categorical variables as frequencies (percentages). Differences across MPO quintiles were examined through ANOVA for continuous variables and χ^2^ tests for categorical measures. Logistic regression models were constructed to evaluate the association between MPO quintiles and anxiety risk using three sequential adjustment strategies: Model 1 adjusted for age and sex; Model 2 additionally incorporated BMI, smoking status, and alcohol consumption; Model 3 further extended adjustments to include education level, occupation type, hypertension, diabetes, and hyperlipidemia. Nonlinear relationships were systematically investigated using restricted cubic splines (RCS) with three knots positioned at the 10th, 50th, and 90th percentiles, complemented by segmented regression analyses to identify potential inflection points. The adequacy of nonlinear versus linear modeling was formally compared through likelihood ratio testing.

To address missing data in occupation and education variables (initially coded as ‘Not recorded’), sensitivity analyses employing multiple imputation by chained equations (MICE) were conducted, with the imputation model encompassing all study variables including age, sex, BMI, lifestyle factors, comorbidities, plasma MPO, and anxiety status. Furthermore, E-value analyses (30) were implemented to quantify the potential bias of unmeasured confounding on the observed associations, estimating the minimum strength required for hypothetical confounders to nullify the MPO-anxiety relationship.

Subgroup analyses were pre-specified to examine potential interaction effects across demographic and clinical strata, including sex, diabetes status, age dichotomization (<45 vs. ≥45 years), BMI categories, smoking behavior, alcohol use patterns, educational attainment, occupational classification, and baseline chronic disease status. Analysis was performed using R 4.2.3^1^ (The R Foundation, Vienna, Austria) and the Free Statistics software (version 2.0; Beijing FreeClinical Medical Technology Co., Ltd., Beijing, China), with statistical significance determined by two-tailed *p*-values < 0.05.

### Baseline characteristics of participants

Among 30,418 Chinese adults included in the analysis, 3,977 individuals (13.07%) were identified with anxiety. Table 1 presents the demographic and clinical characteristics stratified by MPO quintiles. Participants in the highest MPO quintile (Q5: ≥47.3 ng/mL) tended to be younger (42.17 ± 10.86 vs. 45.86 ± 10.01 in Q1, *p* < 0.0001), male (55.35% vs. 54.82% in Q1), and exhibited lower rates of hypertension (14.48% vs. 17.01%) and diabetes (6.32% vs. 8.14%) compared to the lowest quintile (Q1). Notably, anxiety prevalence showed a U-shaped distribution across plasma MPO quintiles (Q1:13.12%, Q2:11.92%, Q3:12.99%, Q4:13.59%, Q5:13.74%). Supplementary Table S2 compares baseline characteristics by anxiety status. Participants with anxiety were more likely to be female (57.23% vs. 44.54%, *p* < 0.0001), engaged in agricultural/industrial occupations (11.92% vs. 6.77%), and had lower educational attainment (college or above: 43.07% vs. 58.43%) and BMI (23.49 vs. 23.79 kg/m^2^, *p* < 0.0001).

**Table 1:** Baseline characteristics of participants by MPO quintiles.

	Total	Q1 (≤29.771)	Q2 (29.8,34.7)	Q3 (34.7,39.7)	Q4 (39.7,47.3)	Q5 (≥ 47.3)	*p*-value
	(*n* = 30,418)	(*n* = 6,115)	(*n* = 6,033)	(*n* = 6,057)	(*n* = 6,121)	(*n* = 6,092)	
Age, years	45.04 ± 10.69	45.86 ± 10.01	46.62 ± 10.58	45.98 ± 10.64	44.57 ± 10.75	42.17 ± 10.86	<0.0001
Sex							<0.01
Female	14,052 (46.20)	2,763 (45.18)	2,801 (46.43)	2,854 (47.12)	2,914 (47.61)	2,720 (44.65)	
Male	16,366 (53.80)	3,352 (54.82)	3,232 (53.57)	3,203 (52.88)	3,207 (52.39)	3,372 (55.35)	
BMI, kg/m^2^	23.75 ± 3.59	23.81 ± 4.00	23.81 ± 3.25	23.77 ± 3.44	23.71 ± 3.53	23.65 ± 3.67	0.07
Smoke							0.35
Current	6,360 (20.91)	1,277 (20.88)	1,245 (20.64)	1,272 (21.00)	1,257 (20.54)	1,309 (21.49)	
Never	22,799 (74.95)	4,550 (74.41)	4,547 (75.37)	4,549 (75.10)	4,621 (75.49)	4,532 (74.39)	
Past	1,259 (4.14)	288 (4.71)	241 (3.99)	236 (3.90)	243 (3.97)	251 (4.12)	
Drink							<0.01
Current	3,454 (11.36)	762 (12.46)	717 (11.88)	712 (11.75)	653 (10.67)	610 (10.01)	
Never	26,725 (87.86)	5,303 (86.72)	5,267 (87.30)	5,303 (87.55)	5,422 (88.58)	5,430 (89.13)	
Past	239 (0.79)	50 (0.82)	49 (0.81)	42 (0.69)	46 (0.75)	52 (0.85)	
Occupation							<0.0001
Agriculture/Industrial	2,264 (7.44)	423 (6.92)	415 (6.88)	446 (7.36)	493 (8.05)	487 (7.99)	
Freelance/Other	11,156 (36.68)	2,180 (35.65)	2,215 (36.71)	2,202 (36.35)	2,276 (37.18)	2,283 (37.48)	
Government/Institution	12,350 (40.60)	2,573 (42.08)	2,386 (39.55)	2,416 (39.89)	2,412 (39.41)	2,563 (42.07)	
Student/Retired	3,042 (10.00)	577 (9.44)	696 (11.54)	673 (11.11)	619 (10.11)	477 (7.83)	
Not record	1,606 (5.28)	362 (5.92)	321 (5.32)	320 (5.28)	321 (5.24)	282 (4.63)	
Education							<0.0001
College or above	17,162 (56.42)	3,505 (57.32)	3,259 (54.02)	3,328 (54.94)	3,442 (56.23)	3,628 (59.55)	
Elementary school or below	3,517 (11.56)	653 (10.68)	779 (12.91)	744 (12.28)	696 (11.37)	645 (10.59)	
Secondary school or vocational school	8,168 (26.85)	1,604 (26.23)	1,682 (27.88)	1,671 (27.59)	1,675 (27.36)	1,536 (25.21)	
Not record	1,571 (5.16)	353 (5.77)	313 (5.19)	314 (5.18)	308 (5.03)	283 (4.65)	
Hypertension							<0.0001
No	25,169 (82.74)	5,075 (82.99)	4,852 (80.42)	4,979 (82.20)	5,053 (82.55)	5,210 (85.52)	
Yes	5,249 (17.26)	1,040 (17.01)	1,181 (19.58)	1,078 (17.80)	1,068 (17.45)	882 (14.48)	
Diabetes							<0.0001
No	28,002 (92.06)	5,617 (91.86)	5,484 (90.90)	5,528 (91.27)	5,666 (92.57)	5,707 (93.68)	
Yes	2,416 (7.94)	498 (8.14)	549 (9.10)	529 (8.73)	455 (7.43)	385 (6.32)	
Hyperlipidemia							<0.01
No	29,916 (98.35)	5,994 (98.02)	5,920 (98.13)	5,967 (98.51)	6,016 (98.28)	6,019 (98.80)	
Yes	502 (1.65)	121 (1.98)	113 (1.87)	90 (1.49)	105 (1.72)	73 (1.20)	
MPO, ng/mL	38.74 ± 12.42	23.82 ± 5.85	32.29 ± 1.40	37.12 ± 1.44	43.15 ± 2.18	57.28 ± 9.83	<0.0001
Anxiety							0.03
No	26,441 (86.93)	5,313 (86.88)	5,314 (88.08)	5,270 (87.01)	5,289 (86.41)	5,255 (86.26)	
Yes	3,977 (13.07)	802 (13.12)	719 (11.92)	787 (12.99)	832 (13.59)	837 (13.74)	

### Association between plasma MPO and risk of anxiety

**Figure 2:**
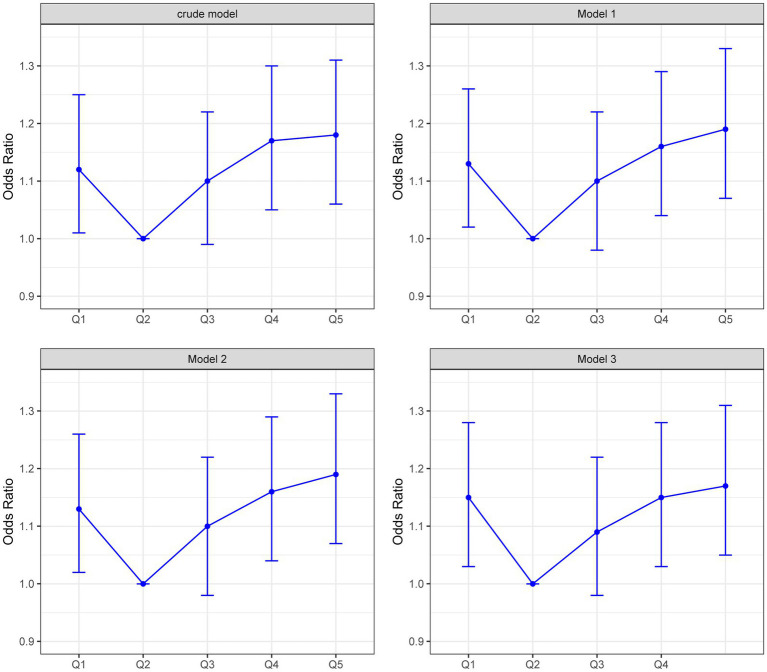
Anxiety risk across MPO quintiles in four regression models.

Logistic regression analyses demonstrated a U-shaped relationship between plasma MPO and anxiety risk (Table 2; Figure 2). In the crude model, participants in the lowest MPO quintile (Q1: ≤29.771 ng/mL) had 12% higher odds of anxiety compared to the reference quintile Q2 (OR = 1.12, 95% CI:1.01–1.25, *p* = 0.03), while those in the highest quintile (Q5: ≥47.3 ng/mL) exhibited an 18% increased risk (OR = 1.18, 95% CI:1.06–1.31, *p* = 0.003). After sequential adjustments for covariates, these associations remained robust: in the fully adjusted model (Model 3), Q1 maintained a 15% elevated risk (OR = 1.15, 95% CI:1.03–1.28, *p* = 0.01), and Q5 retained a 17% increased risk (OR = 1.17, 95% CI:1.05–1.31, *p* = 0.004). Notably, while the third quintile (Q3: 34.7–39.7 ng/mL) showed no statistically significant association (OR = 1.09, 95% CI:0.98–1.22, *p* = 0.10), the fourth quintile (Q4: 39.7–47.3 ng/mL) displayed a consistent risk elevation across all models (Model 3 OR = 1.15, 95% CI:1.03–1.28, p = 0.01). A significant trend toward higher anxiety risk was observed at both extremes of plasma MPO (p for trend = 0.01 in Model 3), further supporting the non-linear nature of this relationship. As detailed in Supplementary Figure S1, missingness was confined to socioeconomic variables with education (5.16%) and occupation (5.28%) showing incomplete records. Distributions of imputed variables showed no significant deviations from complete-case data (Supplementary Table S3), confirming the robustness of our missing data strategy. Sensitivity analyses using multiply imputed datasets (*n* = 5) yielded consistent U-shaped associations between MPO quintiles and anxiety risk. Across all models, the odds ratios for Q1, Q4, and Q5 MPO quintiles remained statistically significant (OR range: 1.14–1.19, all *p* < 0.05), with a non-linear trend (p for trend = 0.002–0.01) mirroring the complete-case findings (Supplementary Table S4). Additionally, E-value analysis indicated that unmeasured confounding would require an OR ≥1.62 to nullify the observed associations, exceeding plausible thresholds for residual bias in this context (Supplementary Figure S2).

**Table 2:** Association between MPO quintiles and anxiety risk.

	Crude model		Model 1		Model 2		Model 3	
	OR (95%CI)	*p*-value	OR (95%CI)	*p*-value	OR (95%CI)	*p-*value	OR (95%CI)	*p*-value
Q2	ref		ref		ref		ref	
Q1	1.12 (1.01,1.25)	0.03	1.13 (1.02,1.26)	0.02	1.13 (1.02,1.26)	0.02	1.15 (1.03,1.28)	0.01
Q3	1.1 (0.99,1.22)	0.08	1.1 (0.98,1.22)	0.10	1.1 (0.98,1.22)	0.10	1.09 (0.98,1.22)	0.10
Q4	1.17 (1.05,1.30)	0.004	1.16 (1.04,1.29)	0.01	1.16 (1.04,1.29)	0.01	1.15 (1.03,1.28)	0.01
Q5	1.18 (1.06,1.31)	0.003	1.19 (1.07,1.33)	0.001	1.19 (1.07,1.33)	0.001	1.17 (1.05,1.31)	0.004
*p* for trend		0.003		0.002		0.002		0.01

Model 1 adjusted for: age, sex. Model 2 adjusted for: age, sex, BMI, smoke, drink. Model 3 adjusted for: age, sex, BMI, smoke, drink, education, occupation, hypertension, diabetes, hyperlipidemia.

### Threshold effect of MPO on anxiety risk

**Figure 3:**
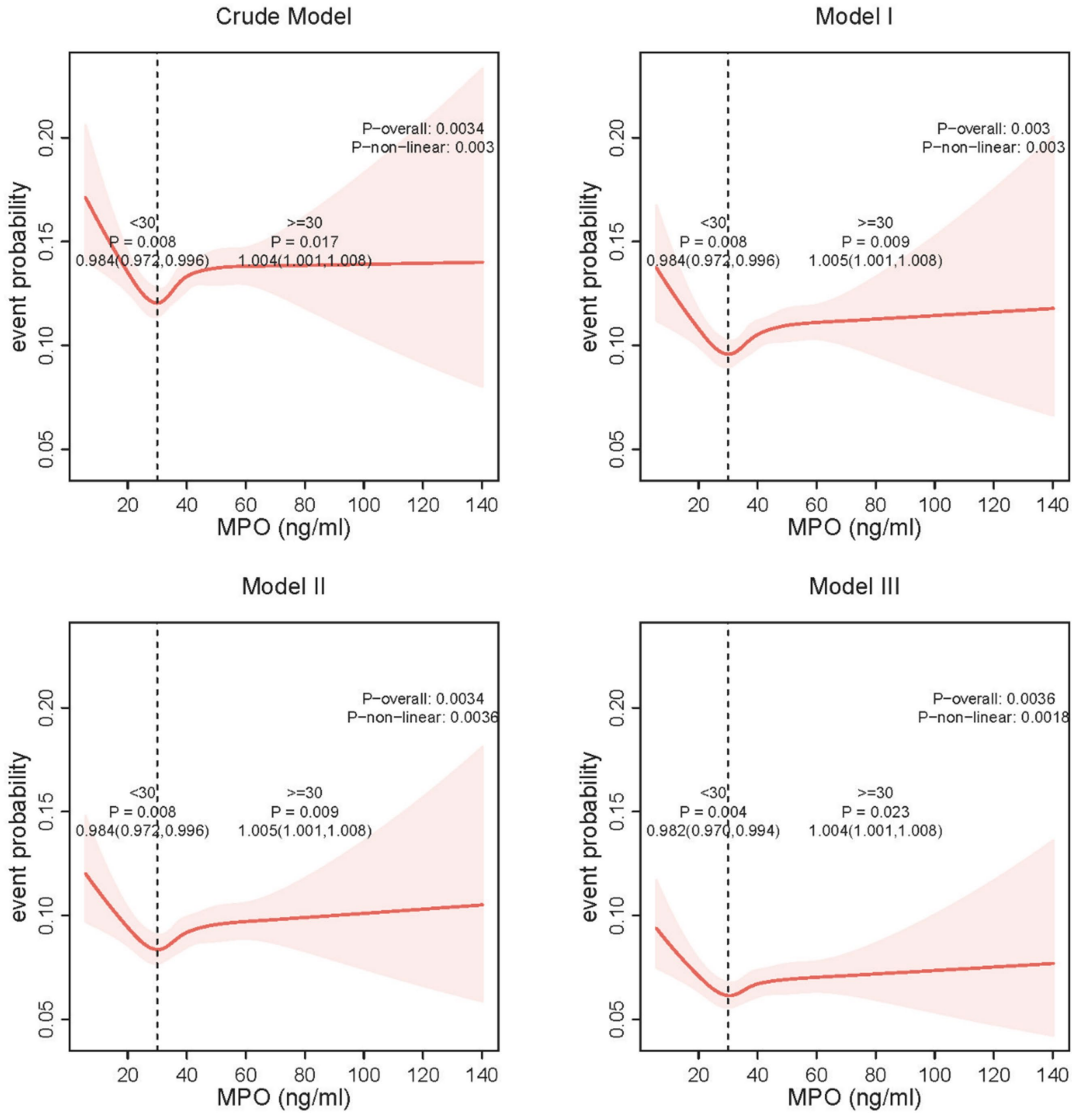
Nonlinear relationship between MPO and anxiety risk.

Restricted cubic spline analysis revealed a U-shaped relationship between plasma MPO and anxiety risk, with a critical inflection point identified at 30 ng/mL (Figure 3). Below this threshold, each 1 ng/mL increase in MPO was associated with reduced anxiety risk, while above 30 ng/mL, higher plasma MPO predicted elevated risk. Detailed threshold effect analyses further quantified these patterns (Table 3). In the crude model, plasma MPO < 30 ng/mL showed a 1.6% reduction in anxiety odds per unit increase (OR = 0.984, 95% CI:0.972–0.996, *p* = 0.008), whereas levels ≥30 ng/mL exhibited a 0.4% risk elevation (OR = 1.004, 95% CI:1.001–1.008, *p* = 0.017). These associations remained remarkably stable across sequential adjustments: in the fully adjusted model (Model 3), the protective effect below 30 ng/mL strengthened (OR = 0.982, 95% CI:0.970–0.994, *p* = 0.004), while the harmful effect above the threshold persisted (OR = 1.004, 95% CI:1.001–1.008, *p* = 0.023). Notably, the two-piecewise linear regression model consistently outperformed the linear assumption in all models (log-likelihood ratio *p* < 0.01), confirming the necessity of modeling MPO’s dual-directional effects. The stability of the inflection point across adjustment stages (30 ng/mL in all models) underscores the robustness of this threshold in defining clinically relevant MPO ranges for anxiety risk stratification.

**Table 3:** Threshold effect analysis of plasma MPO on anxiety risk.

	Crude model	Model 1	Model 2	Model 3
	OR (95%CI) *p*-value	OR (95%CI) *p*-value	OR (95%CI) *p*-value	OR (95%CI) *p*-value
Logistic regression	1.002 (0.999,1.005) 0.151	1.002 (0.999,1.005) 0.126	1.002 (0.999,1.005) 0.121	1.001 (0.999,1.004) 0.373
Two-piecewise linear regression				
MPO < 30	0.984 (0.972,0.996) 0.008	0.984 (0.972,0.996) 0.008	0.984 (0.972,0.996) 0.008	0.982 (0.970,0.994) 0.004
MPO ≥ 30	1.004 (1.001,1.008) 0.017	1.005 (1.001,1.008) 0.009	1.005 (1.001,1.008) 0.009	1.004 (1.001,1.008) 0.023
*p* for Log-likelihood ratio	<0.01	<0.01	<0.01	<0.01

Model 1 adjusted for: age, sex. Model 2 adjusted for: age, sex, BMI, smoke, drink. Model 3 adjusted for: age, sex, BMI, smoke, drink, education, occupation, hypertension, diabetes, hyperlipidemia.

### Subgroup and interaction analyses

Diabetes mellitus (DM) emerged as the sole significant effect modifier in the MPO-anxiety relationship (*p*-interaction = 0.028). Diabetic individuals exhibited a pronounced dose–response pattern, with anxiety risk progressively escalating across MPO quintiles. Compared to non-diabetic counterparts, diabetic participants showed higher risks starting from Q1 (OR = 1.48, 95% CI:0.99–2.21 vs. 1.11, 0.99–1.24), reaching statistical significance in Q3 (OR = 1.63, 1.11–2.42 vs. 1.06, 0.94–1.18), peaking at Q4 (OR = 2.03, 1.38–3.01 vs. 1.11, 0.99–1.24), and remaining elevated in Q5 (OR = 1.84, 1.23–2.78 vs. 1.15, 1.03–1.29). Notably, non-diabetic individuals demonstrated only marginal risk elevation in the highest quintile (Q5 OR = 1.15), whereas diabetic subjects displayed sustained significant risks from Q3 onward. No other subgroups showed meaningful interaction effects (all p-interaction >0.05), highlighting DM’s unique role in potentiating MPO-related neuroinflammatory cascades (Table 4).

**Table 4:** Subgroup analyses and interaction tests for the association between MPO quintiles and anxiety risk.

	Q2	Q1	Q3	Q4	Q5	*p* for interaction
Age_Class						0.921
<45	ref	1.111 (0.937,1.319)	1.023 (0.861,1.216)	1.123 (0.952,1.326)	1.126 (0.961,1.322)	
≥45	ref	1.159 (1.008,1.333)	1.142 (0.994,1.313)	1.188 (1.031,1.369)	1.241 (1.069,1.440)	
Sex						0.528
Female	ref	1.145 (0.990,1.325)	1.139 (0.986,1.315)	1.126 (0.975,1.301)	1.235 (1.067,1.429)	
Male	ref	1.117 (0.951,1.313)	1.031 (0.874,1.217)	1.203 (1.025,1.414)	1.121 (0.954,1.318)	
BMI						0.92
(24, 28)	ref	1.123 (0.933,1.353)	1.128 (0.937,1.358)	1.161 (0.964,1.398)	1.167 (0.966,1.410)	
<24	ref	1.109 (0.961,1.281)	1.074 (0.930,1.240)	1.139 (0.988,1.313)	1.203 (1.044,1.388)	
≥28	ref	1.326 (0.932,1.893)	1.058 (0.738,1.520)	1.226 (0.865,1.745)	1.090 (0.765,1.558)	
Smoke						0.292
Never	ref	1.148 (1.014,1.299)	1.130 (0.999,1.278)	1.166 (1.031,1.319)	1.221 (1.079,1.383)	
Current	ref	1.190 (0.936,1.516)	1.035 (0.809,1.324)	1.129 (0.885,1.441)	1.164 (0.916,1.481)	
Past	ref	0.675 (0.374,1.208)	0.736 (0.400,1.335)	1.261 (0.737,2.173)	0.687 (0.373,1.246)	
Drink						0.835
Never	ref	1.122 (0.999,1.259)	1.123 (1.002,1.260)	1.170 (1.044,1.312)	1.205 (1.074,1.351)	
Current	ref	1.222 (0.890,1.684)	0.903 (0.641,1.269)	1.065 (0.762,1.490)	1.105 (0.788,1.550)	
Past	ref	1.132 (0.364,3.580)	0.858 (0.233,2.965)	1.662 (0.545,5.258)	0.865 (0.268,2.775)	
Occupation						0.527
Not record	ref	1.217 (0.776, 1.921)	1.171 (0.734,1.875)	1.240 (0.781,1.979)	1.101 (0.673,1.802)	
Freelance/Other	ref	1.136 (0.959,1.347)	0.947 (0.795,1.128)	1.169 (0.989,1.383)	1.051 (0.885,1.248)	
Government/Institution	ref	1.174 (0.969,1.423)	1.227 (1.013,1.488)	1.198 (0.987,1.454)	1.357 (1.125,1.639)	
Student/Retired	ref	1.092 (0.790,1.508)	1.170 (0.862,1.592)	0.903 (0.649,1.255)	1.099 (0.777,1.549)	
Agriculture/Industrial	ref	1.062 (0.753,1.498)	1.114 (0.799,1.555)	1.136 (0.821,1.577)	1.168 (0.843,1.623)	
Education						0.909
Not record	ref	1.207 (0.770,1.909)	1.157 (0.725, 1.854)	1.233 (0.774, 1.972)	1.103 (0.677, 1.801)	
Secondary school or vocational school	ref	1.286 (1.072,1.545)	1.086 (0.903,1.308)	1.174 (0.977,1.411)	1.136 (0.940,1.373)	
College or above	ref	1.090 (0.924,1.287)	1.120 (0.949,1.323)	1.143 (0.971,1.347)	1.184 (1.009,1.392)	
Elementary school or below	ref	1.065 (0.806,1.404)	1.020 (0.781,1.330)	1.120 (0.857,1.464)	1.257 (0.959,1.647)	
Hypertension						0.066
No	ref	1.138 (1.011,1.282)	1.038 (0.920,1.172)	1.182 (1.050,1.330)	1.181 (1.049,1.330)	
Yes	ref	1.094 (0.844,1.418)	1.373 (1.074,1.758)	1.059 (0.817,1.371)	1.260 (0.967,1.639)	
Diabetes						0.028
No	ref	1.106 (0.989,1.238)	1.057 (0.944,1.183)	1.107 (0.990,1.238)	1.149 (1.027,1.285)	
Yes	ref	1.479 (0.994,2.210)	1.634 (1.113,2.416)	2.032 (1.384,3.007)	1.841 (1.226,2.776)	
Hyperlipidemia						0.328
No	ref	1.124 (1.008,1.253)	1.082 (0.970,1.206)	1.150 (1.032,1.281)	1.188 (1.066,1.324)	
Yes	ref	1.772 (0.731, 4.593)	2.515 (1.031, 6.565)	2.413 (1.007, 6.224)	1.305 (0.432, 3.850)	

Adjusted for: age, sex, BMI, smoke, drink, education, occupation, hypertension, diabetes, hyperlipidemia except the modifier.

## Discussion

Our objective is to investigate whether plasma MPO is associated with anxiety risk in Chinese adults. Since MPO can regulate neuroinflammatory responses, and neuroinflammation is considered a key feature of mental disorders such as anxiety symptoms, exploring the relationship between MPO and anxiety is of significant importance. To our knowledge, this is the first large-scale population-based study in Chinese adults that explores the association between MPO and anxiety after adjusting for confounding factors. Our findings reveal that the relationship between plasma MPO and anxiety risk is not linear but instead follows a U-shaped pattern. Additionally, we identified that diabetes may exacerbate MPO-related anxiety risk. After comprehensive adjustment for confounding variables, individuals in both the lowest (Q1 ≤ 29.77 ng/mL) and highest (Q5 ≥ 47.3 ng/mL) MPO quintiles exhibited significantly increased anxiety risk. Furthermore, we identified an MPO concentration of 30 ng/mL as a critical threshold: below this level, MPO appears to exert a protective effect, whereas levels exceeding this threshold are associated with an increased risk of anxiety. These findings suggest that MPO plays a dual role in anxiety regulation, depending on its concentration.

Notably, although our study also found a higher overall prevalence of anxiety among women, sex did not significantly modify the association between MPO levels and anxiety risk. In interaction analyses, the relationship between MPO levels and anxiety risk remained consistent across both males and females, indicating that the anxiety-related effects of MPO are uniform between sexes. The higher anxiety prevalence observed in women is more likely driven by psychosocial factors—such as gender role expectations and exposure to stress (31)—rather than differences in biological markers like MPO. Therefore, sex does not appear to influence the role of MPO in the pathogenesis of anxiety.

As a key regulator of immune responses and oxidative stress, the U-shaped relationship between plasma MPO and anxiety risk may be closely linked to its biological functions. MPO plays a dual role in immune responses (32). On one hand, MPO contributes to neutrophil antibacterial activity and enhances the body’s defense against various pathogens (33), thereby supporting innate immune responses (34). Notably, immune deficiencies have been reported to be associated with anxiety, sleep disorders, and other psychiatric conditions (35, 36). This suggests that immune imbalance caused by low plasma MPO may contribute to anxiety development, although further research is needed to validate this hypothesis. On the other hand, elevated plasma MPO have been implicated in the progression of various chronic inflammatory diseases (37), and chronic inflammation is known to significantly increase the risk of anxiety (38). Thus, MPO is neither purely a protective nor a pathogenic factor; its function largely depends on its expression level. Interestingly, our subgroup analysis showed that participants in the highest MPO quintile (Q5) were, on average, younger and had lower prevalence of chronic conditions such as diabetes and hypertension. This observation appears paradoxical, as chronic diseases are often associated with elevated MPO levels and systemic inflammation. One possible explanation is that elevated MPO in these individuals may reflect early subclinical inflammation preceding overt disease onset. Previous studies have shown that elevated MPO levels in prepubertal obese children are associated with pro-inflammatory and cardiovascular risk biomarkers such as C-reactive protein (CRP), MMP-9, and resistin, suggesting that MPO may serve as an early inflammatory biomarker related to cardiovascular risk in this population (39). Since CRP is considered a marker of chronic low-grade inflammation (40), MPO may contribute to the development of chronic diseases by influencing low-grade inflammatory responses. Therefore, high MPO levels in apparently healthy individuals might serve as an early biomarker of low-grade inflammatory risk, potentially linking subclinical immune activation to the emergence of inflammatory diseases. This finding expands the potential utility of MPO, suggesting that it may serve not only as a marker of established inflammatory diseases but also as a candidate indicator for diseases associated with chronic low-grade inflammation. Our study also identified that diabetes may exacerbate MPO-induced anxiety risk, which could be attributed to the significantly elevated plasma MPO observed in type 2 diabetes patients (41, 42). Diabetes-induced MPO elevation likely triggers chronic inflammation, intensifying neuroinflammatory responses. Additionally, a recent review highlighted that diabetes is often accompanied by dysregulation of the hypothalamic–pituitary–adrenal axis, a well-established contributor to anxiety disorders (43). This suggests that molecular pathophysiological mechanisms underlying diabetes progression may further facilitate the onset of anxiety.

Previous studies on the relationship between MPO and psychiatric disorders have primarily focused on its association with depression. MPO activity has been shown to increase during depressive episodes (44). Compared to control groups, patients with depression exhibit significantly elevated MPO expression at both the mRNA and protein levels, highlighting its crucial role in cognitive function regulation (45). In an evaluation of the antidepressant and antioxidant effects of quetiapine in rats, its administration was found to be accompanied by MPO activity inhibition (46). Notably, MPO inhibition has been proposed as a potential therapeutic strategy for treating inflammation-associated major depressive disorder (47). These findings suggest that MPO plays a promotive role in the pathophysiology of depression. Additionally, a previous study reported increased MPO activity in mice exhibiting both depression-like and anxiety-like behaviors (26), suggesting a possible link between MPO and anxiety. Our study confirms that variations in plasma MPO influence anxiety risk; however, rather than a conventional linear relationship, we identified a U-shaped association. Specifically, maintaining plasma MPO at approximately 30 ng/mL appears to be optimal, as both higher and lower levels are significantly associated with anxiety. This finding implies that moderate MPO levels may help balance pathogen clearance and neuroimmune homeostasis, thereby mitigating pathological processes related to anxiety.

Although our study demonstrates a significant U-shaped association between MPO and anxiety risk, it is worth noting that the diagnostic utility of MPO as a standalone biomarker may be limited. Our ROC analysis yielded an AUC of 0.51 (95% CI: 0.50–0.52), with an optimal cutoff value of 39.38 ng/mL, corresponding to a sensitivity of 43.2% and specificity of 59.1% (data not shown). The low discriminatory performance may stem from the non-linear (U-shaped) relationship between MPO and anxiety, which differs from the typically linear association seen in other inflammatory markers.

For example, IL-17A and IL-23A, two proinflammatory cytokines associated with Th17 activation, have shown promise as biomarkers for generalized anxiety disorder (GAD). A study reported significantly elevated serum IL-17A and IL-23A levels in GAD patients, with AUC values of 0.710 and 0.824, respectively, and corresponding sensitivities of 77.27% and 80.49% (48). These findings suggest that IL-17A and IL-23A may have superior predictive power compared to MPO for identifying anxiety in clinical settings. However, MPO may still offer practical value in population-level risk stratification. In our study, individuals in both the lowest and highest MPO quintiles had approximately 15%–17% increased risk of anxiety compared to the moderate group. This suggests that plasma MPO may serve as a useful early warning indicator, especially when combined with demographic factors such as female sex or lower educational attainment. While MPO may lack diagnostic precision, its ease of measurement through routine blood testing and its ability to identify vulnerable subpopulations highlight its potential in large-scale screening or as part of multi-marker predictive models.

Although this study reveals a significant association between plasma MPO and anxiety risk, several limitations should be acknowledged. First, the cross-sectional design precludes causal inference, and thus we cannot determine whether changes in MPO levels precede or result from anxiety onset. It is also important to note the possibility of reverse causality, where elevated anxiety symptoms could influence plasma MPO levels, rather than the other way around. Given the nature of our study, we cannot establish the directionality of the association, and the observed relationship may reflect bidirectional or reverse causal pathways. Longitudinal cohort studies and interventional trials are warranted to establish causality and disentangle the temporal sequence. Second, only peripheral plasma MPO was measured, which may not directly reflect MPO activity within the central nervous system. Future investigations incorporating cerebrospinal fluid analysis or neuroimaging could offer a more comprehensive understanding of the neuroinflammatory mechanisms involved. Third, other key inflammatory markers were not included in our analyses, limiting our ability to compare MPO’s role within the broader inflammatory profile. While multiple strategies were applied to mitigate potential biases, certain methodological concerns remain. For variables with missing data (i.e., occupation and education), we employed multiple imputation under the assumption of missing at random (MAR). Although the distributions of imputed variables closely mirrored those of the complete-case data (Supplementary Table S3), residual bias may persist if missingness was related to unmeasured socioeconomic factors. To assess the robustness of our findings, we performed E-value analysis, which showed that an unmeasured confounder would need to have a risk ratio of at least 1.66 (for the highest MPO quintile, OR = 1.17) with both the exposure and outcome to fully explain away the observed association—an effect size that is unlikely in this context. Lastly, anxiety status was assessed using the SAS, a validated and widely used screening tool in epidemiological studies. However, as a self-reported measure, it is susceptible to recall and reporting bias, potentially leading to misclassification of anxiety severity or status. Although the SAS demonstrates good internal consistency in Chinese populations, future studies should consider incorporating structured clinical interviews to enhance diagnostic precision. Developing multi-biomarker predictive models in future studies may improve the accuracy of anxiety risk assessment. To address these limitations, future research should employ longitudinal cohort studies to investigate the temporal dynamics between plasma MPO and anxiety development. Furthermore, animal models and cellular experiments could help elucidate the neuroinflammatory and oxidative stress mechanisms underlying MPO-mediated anxiety. Evaluating the potential application of antioxidant and anti-inflammatory strategies in MPO-related anxiety management is also warranted. Notably, diabetic patients should be particularly aware of the heightened anxiety risk associated with MPO dysregulation, and anti-inflammatory approaches could be considered as adjunctive interventions to facilitate personalized treatment strategies. Although this study is based on a nationally representative sample of Chinese adults, caution is warranted when generalizing the findings to other populations. Variations in genetic background may affect both MPO levels and the risk of mental disorders (49, 50). Future research involving diverse international cohorts is essential to confirm the generalizability of these results across varying demographic and clinical contexts.

In conclusion, this study is the first to reveal a U-shaped relationship between plasma MPO and anxiety risk and to identify diabetes as a factor that exacerbates MPO-related anxiety risk. These findings provide new evidence supporting MPO as a predictive biomarker for anxiety and suggest potential inflammation-targeted interventions for anxiety management. Future research should further explore MPO-related neuroinflammatory mechanisms and assess the role of personalized anti-inflammatory therapies in anxiety treatment.

## Data Availability

The raw data supporting the conclusions of this article will be made available by the authors, without undue reservation.

## Footnotes

^1^ http://www.Rproject.org
