# Supplementary material for: U-shaped association between myeloperoxidase levels and anxiety risk: a cross-sectional study in a Chinese population
Source: Front Public Health. 2025 May 7;13:1596844. doi: 10.3389/fpubh.2025.1596844 (PMC12092346; doi:10.3389/fpubh.2025.1596844)
Supplement: Supplementary file 1 [file Data_Sheet_1.docx]

***Supplementary Material***

**U-Shaped Association Between Myeloperoxidase Levels and Anxiety Risk: A Cross-Sectional Study in a Chinese Population**

Junteng Zhou^1#^, Qihang Kong^2#^, Xiaojing Liu^2,3**^ and Yan Huang^1,4,5,6*^

^1^Health Management Center, General Practice Medical Center, West China Hospital, Sichuan University, Chengdu 610041, China

^2^Laboratory of Cardiovascular Diseases, Regenerative Medicine Research Center, West China Hospital, Sichuan University, Chengdu, China.

^3^Department of Cardiology, West China Hospital, Sichuan University, Chengdu, China

^4^State Key Laboratory of Respiratory Health and Multimorbidity

^5^Research Laboratory for Prediction and Evaluation of Chronic Diseases in the Elderly, National Clinical Research Center for Geriatric Diseases

^6^General Practice Research Institute, West China Hospital, Sichuan University, Chengdu, China.

*Correspondence to: Yan Huang, Health Management Center, General Practice Medical Center, West China Hospital, Sichuan University, Chengdu 610041, China.

E-mail: yanhuang@wchscu.cn;

**Xiaojing Liu, Laboratory of Cardiovascular Diseases, West China Hospital, Sichuan University, Chengdu, Sichuan Province, 610041, PR China.

E-mail: liuxq@scu.edu.cn.

#Junteng Zhou and Qihang Kong contributed equally to this work.


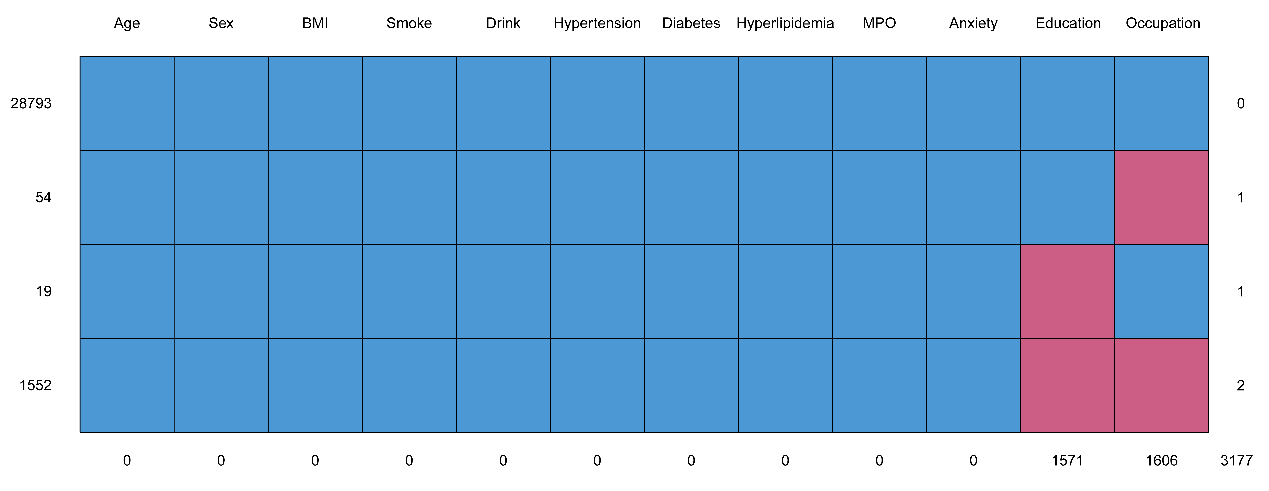


Figure S1. Missing distrubution of variables in the study.


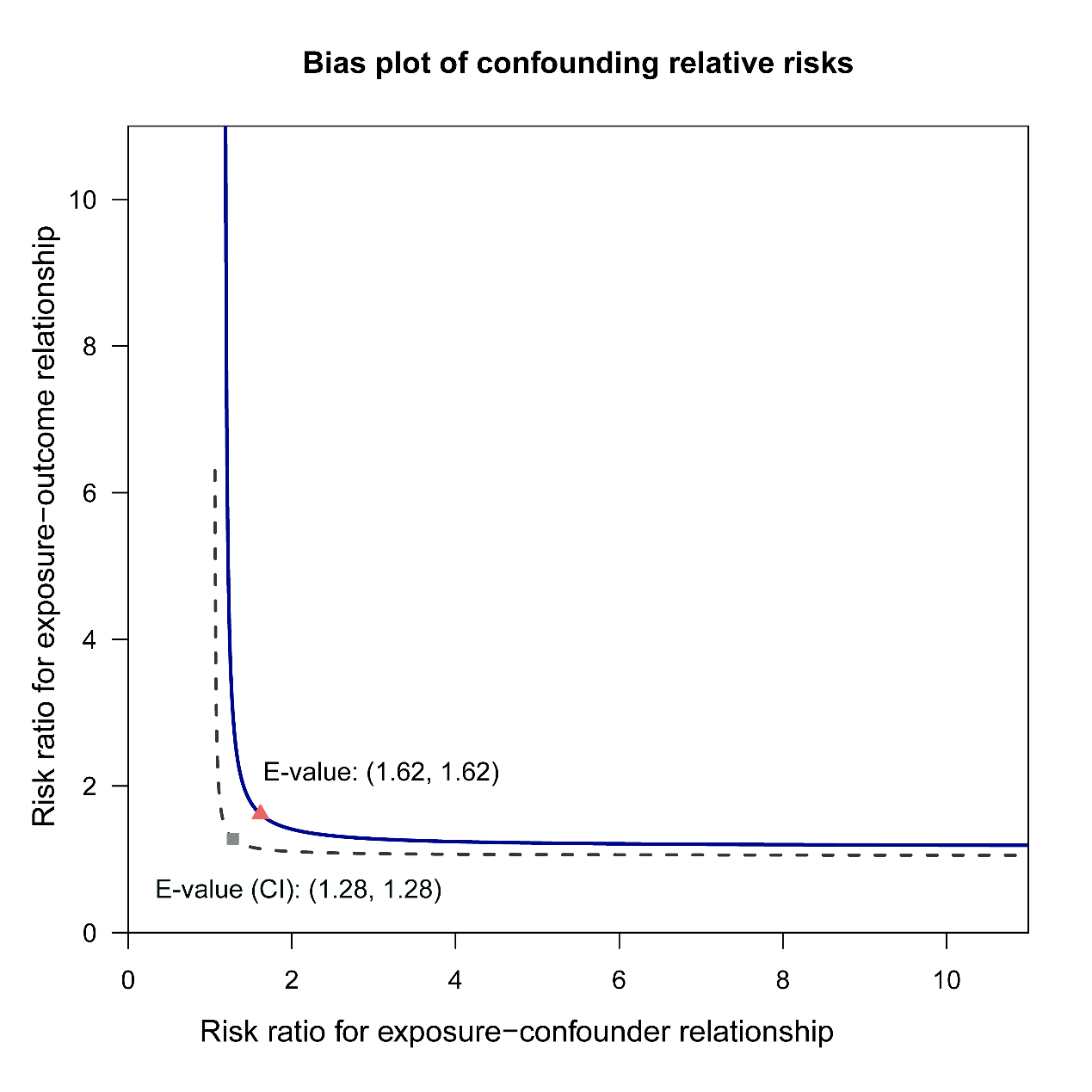


Figure S2. Value of the joint minimum strength of association on the risk ratio scale that an unmeasured confounder would be required to have with plasma MPO (the exposure), and anxiety risk (the outcome).

Table S1. Characteristics of the participants excluded from the analyses and those included in the final analyses.

|  | Total (n=63564) | Excluded (n=33146) | Included (n=30418) |
| --- | --- | --- | --- |
| Age,years | 46.41 ± 12.03 | 47.67 ± 13.01 | 45.04 ± 10.69 |
| Sex |  |  |  |
| Female | 29799(46.88) | 15747(47.51) | 14052(46.20) |
| Male | 33765(53.12) | 17399(52.49) | 16366(53.80) |
| BMI,kg/m2 | 23.65 ± 3.48 | 23.56 ± 3.36 | 23.75 ± 3.59 |
| Smoke |  |  |  |
| Current | 12106(19.05) | 5746(17.34) | 6360(20.91) |
| Never | 48809(76.79) | 26010(78.47) | 22799(74.95) |
| Past | 2649( 4.17) | 1390( 4.19) | 1259( 4.14) |
| Drink |  |  |  |
| Current | 6137( 9.65) | 2683( 8.09) | 3454(11.36) |
| Never | 56910(89.53) | 30185(91.07) | 26725(87.86) |
| Past | 517( 0.81) | 278( 0.84) | 239( 0.79) |
| Occupation |  |  |  |
| Agriculture/Industrial | 4223( 6.64) | 1959( 5.91) | 2264( 7.44) |
| Freelance/Other | 18626(29.30) | 7470(22.54) | 11156(36.68) |
| Government/Institution | 30023(47.23) | 17673(53.32) | 12350(40.60) |
| Not record | 3233( 5.09) | 1627( 4.91) | 1606( 5.28) |
| Student/Retired | 7459(11.73) | 4417(13.33) | 3042(10.00) |
| Education |  |  |  |
| College or above | 38286(60.23) | 21124(63.73) | 17162(56.42) |
| Elementary school or below | 6822(10.73) | 3305( 9.97) | 3517(11.56) |
| Not record | 3184( 5.01) | 1613( 4.87) | 1571( 5.16) |
| Secondary school or vocational school | 15272(24.03) | 7104(21.43) | 8168(26.85) |
| Hypertension |  |  |  |
| No | 52013(81.83) | 26844(80.99) | 25169(82.74) |
| Yes | 11551(18.17) | 6302(19.01) | 5249(17.26) |
| Diabetes |  |  |  |
| No | 58446(91.95) | 30444(91.85) | 28002(92.06) |
| Yes | 5118( 8.05) | 2702( 8.15) | 2416( 7.94) |
| Hyperlipidemia |  |  |  |
| No | 62522(98.36) | 32606(98.37) | 29916(98.35) |
| Yes | 1042( 1.64) | 540( 1.63) | 502( 1.65) |
| MPO,ng/mL | 38.31 ± 12.33 | 37.86 ± 12.21 | 38.74 ± 12.42 |

Table S2. Baseline characteristics of participants by Anxiety status.

|  | Total (n=30418) | Without Anxiety (n=26441) | With Anxiety (n=3977) | p.value |
| --- | --- | --- | --- | --- |
| Age,years | 45.04 ± 10.69 | 45.05 ± 10.62 | 44.93 ± 11.11 | 0.51 |
| Sex |  |  |  | <0.0001 |
| Female | 14052(46.20) | 11776(44.54) | 2276(57.23) |  |
| Male | 16366(53.80) | 14665(55.46) | 1701(42.77) |  |
| BMI,kg/m2 | 23.75 ± 3.59 | 23.79 ± 3.61 | 23.49 ± 3.40 | <0.0001 |
| Smoke |  |  |  | <0.001 |
| Current | 6360(20.91) | 5566(21.05) | 794(19.96) |  |
| Never | 22799(74.95) | 19743(74.67) | 3056(76.84) |  |
| Past | 1259( 4.14) | 1132( 4.28) | 127( 3.19) |  |
| Drink |  |  |  | 0.04 |
| Current | 3454(11.36) | 3048(11.53) | 406(10.21) |  |
| Never | 26725(87.86) | 23190(87.70) | 3535(88.89) |  |
| Past | 239( 0.79) | 203( 0.77) | 36( 0.91) |  |
| Occupation |  |  |  | <0.0001 |
| Agriculture/Industrial | 2264( 7.44) | 1790( 6.77) | 474(11.92) |  |
| Freelance/Other | 11156(36.68) | 9572(36.20) | 1584(39.83) |  |
| Government/Institution | 12350(40.60) | 11072(41.87) | 1278(32.13) |  |
| Not record | 1606( 5.28) | 1386( 5.24) | 220( 5.53) |  |
| Student/Retired | 3042(10.00) | 2621( 9.91) | 421(10.59) |  |
| Education |  |  |  | <0.0001 |
| College or above | 17162(56.42) | 15449(58.43) | 1713(43.07) |  |
| Elementary school or below | 3517(11.56) | 2870(10.85) | 647(16.27) |  |
| Not record | 1571( 5.16) | 1351( 5.11) | 220( 5.53) |  |
| Secondary school or vocational school | 8168(26.85) | 6771(25.61) | 1397(35.13) |  |
| Hypertension |  |  |  | 0.79 |
| No | 25169(82.74) | 21872(82.72) | 3297(82.90) |  |
| Yes | 5249(17.26) | 4569(17.28) | 680(17.10) |  |
| Diabetes |  |  |  | 0.77 |
| No | 28002(92.06) | 24346(92.08) | 3656(91.93) |  |
| Yes | 2416( 7.94) | 2095( 7.92) | 321( 8.07) |  |
| Hyperlipidemia |  |  |  | 0.58 |
| No | 29916(98.35) | 26000(98.33) | 3916(98.47) |  |
| Yes | 502( 1.65) | 441( 1.67) | 61( 1.53) |  |

Table S3. Distributions of variables comparing observed complete case data to results from the datasets with imputed variables from multiple imputation.

|  | Complete case | Multi Imputation |  |  |  |  |
| --- | --- | --- | --- | --- | --- | --- |
|  |  | 1 (n=30418) | 2 (n=30418) | 3 (n=30418) | 4 (n=30418) | 5 (n=30418) |
| Age | 45.04 ± 10.69 | 45.04 ± 10.69 | 45.04 ± 10.69 | 45.04 ± 10.69 | 45.04 ± 10.69 | 45.04 ± 10.69 |
| Sex |  |  |  |  |  |  |
| Female | 14052(46.20) | 14052(46.20) | 14052(46.20) | 14052(46.20) | 14052(46.20) | 14052(46.20) |
| Male | 16366(53.80) | 16366(53.80) | 16366(53.80) | 16366(53.80) | 16366(53.80) | 16366(53.80) |
| BMI | 23.75 ± 3.59 | 23.75 ± 3.59 | 23.75 ± 3.59 | 23.75 ± 3.59 | 23.75 ± 3.59 | 23.75 ± 3.59 |
| Smoke |  |  |  |  |  |  |
| CURRENT | 6360(20.91) | 6360(20.91) | 6360(20.91) | 6360(20.91) | 6360(20.91) | 6360(20.91) |
| NEVER | 22799(74.95) | 22799(74.95) | 22799(74.95) | 22799(74.95) | 22799(74.95) | 22799(74.95) |
| PAST | 1259( 4.14) | 1259( 4.14) | 1259( 4.14) | 1259( 4.14) | 1259( 4.14) | 1259( 4.14) |
| Drink |  |  |  |  |  |  |
| CURRENT | 3454(11.36) | 3454(11.36) | 3454(11.36) | 3454(11.36) | 3454(11.36) | 3454(11.36) |
| NEVER | 26725(87.86) | 26725(87.86) | 26725(87.86) | 26725(87.86) | 26725(87.86) | 26725(87.86) |
| PAST | 239( 0.79) | 239( 0.79) | 239( 0.79) | 239( 0.79) | 239( 0.79) | 239( 0.79) |
| Occupation |  |  |  |  |  |  |
| Agriculture/Industrial | 2264( 7.86) | 2423( 7.97) | 2402( 7.90) | 2406( 7.91) | 2403( 7.90) | 2394( 7.87) |
| Freelance/Other | 11156(38.72) | 11751(38.63) | 11750(38.63) | 11748(38.62) | 11813(38.84) | 11757(38.65) |
| Government/Institution | 12350(42.86) | 13028(42.83) | 13031(42.84) | 13039(42.87) | 12987(42.70) | 13046(42.89) |
| Student/Retired | 3042(10.56) | 3216(10.57) | 3235(10.64) | 3225(10.60) | 3215(10.57) | 3221(10.59) |
| Education |  |  |  |  |  |  |
| College or above | 17162(59.49) | 18050(59.34) | 18074(59.42) | 18065(59.39) | 18039(59.30) | 18074(59.42) |
| Elementary school or below | 3517(12.19) | 3732(12.27) | 3717(12.22) | 3713(12.21) | 3727(12.25) | 3707(12.19) |
| Secondary school or vocational school | 8168(28.31) | 8636(28.39) | 8627(28.36) | 8640(28.40) | 8652(28.44) | 8637(28.39) |
| Hypertension |  |  |  |  |  |  |
| No | 25169(82.74) | 25169(82.74) | 25169(82.74) | 25169(82.74) | 25169(82.74) | 25169(82.74) |
| Yes | 5249(17.26) | 5249(17.26) | 5249(17.26) | 5249(17.26) | 5249(17.26) | 5249(17.26) |
| Diabetes |  |  |  |  |  |  |
| No | 28002(92.06) | 28002(92.06) | 28002(92.06) | 28002(92.06) | 28002(92.06) | 28002(92.06) |
| Yes | 2416( 7.94) | 2416( 7.94) | 2416( 7.94) | 2416( 7.94) | 2416( 7.94) | 2416( 7.94) |
| Hyperlipidemia |  |  |  |  |  |  |
| No | 29916(98.35) | 29916(98.35) | 29916(98.35) | 29916(98.35) | 29916(98.35) | 29916(98.35) |
| Yes | 502( 1.65) | 502( 1.65) | 502( 1.65) | 502( 1.65) | 502( 1.65) | 502( 1.65) |
| MPO | 38.74 ± 12.42 | 38.74 ± 12.42 | 38.74 ± 12.42 | 38.74 ± 12.42 | 38.74 ± 12.42 | 38.74 ± 12.42 |
| Anxiety |  |  |  |  |  |  |
| No | 26441(86.93) | 26441(86.93) | 26441(86.93) | 26441(86.93) | 26441(86.93) | 26441(86.93) |
| Yes | 3977(13.07) | 3977(13.07) | 3977(13.07) | 3977(13.07) | 3977(13.07) | 3977(13.07) |

Table S4. Effect of MPO on anxiety from the datasets with imputed variables from multiple imputation.

|  | crude model |  | Model 1 |  | Model 2 |  | Model 3 |  |
| --- | --- | --- | --- | --- | --- | --- | --- | --- |
|  | OR (95%CI) | p.value | OR (95%CI) | p.value | OR (95%CI) | p.value | OR (95%CI) | p.value |
|  |  |  |  |  |  |  |  |  |
| Q2 | ref |  | ref |  | ref |  | ref |  |
| Q1 | 1.12(1.00,1.24) | 0.05 | 1.12(1.01,1.25) | 0.03 | 1.12(1.01,1.25) | 0.03 | 1.15(1.03,1.28) | 0.01 |
| Q3 | 1.1(0.99,1.23) | 0.07 | 1.1(0.99,1.23) | 0.08 | 1.1(0.99,1.23) | 0.09 | 1.1(0.99,1.23) | 0.09 |
| Q4 | 1.16(1.04,1.29) | 0.01 | 1.16(1.04,1.29) | 0.01 | 1.16(1.04,1.29) | 0.01 | 1.14(1.03,1.27) | 0.02 |
| Q5 | 1.18(1.06,1.31) | 0.003 | 1.19(1.07,1.33) | 0.002 | 1.19(1.07,1.33) | 0.002 | 1.17(1.05,1.31) | 0.004 |
| p for trend |  | 0.003 |  | 0.002 |  | 0.002 |  | 0.01 |

|  | |  |  |  |  |  |  |
| --- | --- | --- | --- | --- | --- | --- | --- |
| Model 1 adjusted for : Age, Sex | | | | | | | |
| Model 2 adjusted for : Age, Sex, BMI, Smoke, Drink | | | | | | | |
| Model 3 adjusted for : Age, Sex, BMI, Smoke, Drink, Education, Occupation, Hypertension, Diabetes, Hyperlipidemia | | | | | | | |
